# Next Location Prediction Based on an Adaboost-Markov Model of Mobile Users †

**DOI:** 10.3390/s19061475

**Published:** 2019-03-26

**Authors:** Hongjun Wang, Zhen Yang, Yingchun Shi

**Affiliations:** National University of Defense Technology, Hefei 230037, China; mielideman@163.com (H.W.); ycshi_eei@163.com (Y.S.)

**Keywords:** next location prediction, trajectory division, important locations, density clustering, Markov model, Adaboost algorithm

## Abstract

As an emerging class of spatial trajectory data, mobile user trajectory data can be used to analyze individual or group behavioral characteristics, hobbies and interests. Besides, the information extracted from original trajectory data is widely used in smart cities, transportation planning, and anti-terrorism maintenance. In order to identify the important locations of the target user from his trajectory data, a novel division method for preprocessing trajectory data is proposed, the feature points of original trajectory are extracted according to the change of trajectory structural, and then important locations are extracted by clustering the feature points, using an improved density peak clustering algorithm. Finally, in order to predict next location of mobile users, a multi-order fusion Markov model based on the Adaboost algorithm is proposed, the model order *k* is adaptively determined, and the weight coefficients of the 1~*k*-order models are given by the Adaboost algorithm according to the importance of various order models, a multi-order fusion Markov model is generated to predict next important location of the user. The experimental results on the real user trajectory dataset Geo-life show that the prediction performance of Adaboost-Markov model is better than the multi-order fusion Markov model with equal coefficient, and the universality and prediction performance of Adaboost-Markov model is better than the first to third order Markov models.

## 1. Introduction

In recent years, with the rapid development of mobile communication technology and the increasingly powerful functions of smart mobile terminal networks, smart terminal devices such as mobile phones and tablet computers have gradually surpassed personal computers and become the most widely used information devices for people. At the same time, the rapid development of global navigation and positioning systems has provided accurate and real-time location information for smart mobile terminals [1]. Location-based services (LBSs) have become some of the most popular terminal information services, and a large number of related studies have also proved the hidden value of mining the resulting massive location data. Currently, location-based services mainly include location query, path planning, location sharing, etc. These functions are mostly focused on providing users with relevant services at the current location. In order to make the service more forward-looking, in recent years, a large number of domestic and foreign scholars have turned their attention to the location prediction of mobile users [2,3,4,5]. Location prediction concentrates on predicting the next location of mobile users with maximum likelihood, on the basis of the user’s past trajectory data. The current sources of trajectory data are mainly the following: (1) Data collected by the sensors, such as Global Positioning System (GPS) [6,7,8,9], wireless fidelity (Wi-Fi) sensors [10,11,12,13,14] and base stations [15,16,17]. (2) Users’ check-in data on social software [18,19], in recent years, with the promotion of location-based social networks, a large number of check-in data with time and space tags have been generated. (3) Vehicle traffic data recorded by city traffic bayonets [20,21].

Mobile user location prediction has high research value and broad application prospects, as research shows that human activities have a strong regularity, and the potential predictability of user mobility is 93% [22]. The most intuitive applications include: (1) personalized recommendations and reminders [23,24,25]: according to the predicted next location of the user, personalized recommendations and reminders can be provided to the user. For example, the system predicts that a user’s next location is a bar, the promotion of the bar and some new “friends come here” messages can be recommended to the user; (2) suspicious target tracking [26,27]: mobile user location prediction can be used to track and locate suspicious users, such as tracking users who make extreme comments on the Internet. It can effectively prevent the occurrence of events that may endanger public safety by predicting the future destination of the target users; (3) intelligent transportation [28]: a large amount of trajectory data collected by vehicle GPS devices can be used to predict traffic congestion, so that drivers can plan their driving routes and improve the scheduling efficiency of taxis.

At the same time, mobile user location prediction is also a very difficult and challenging task. First of all, due to the occlusion of high buildings in the city and the user’s random switching device positioning function, it is easy to cause the loss of some important data and there is a lack of training data. Second, the activities of mobile users are uncertain, and the mobile modes exhibited by the same user may be quite different in different time periods. Furthermore, the user’s trajectory data is both discrete and massive, and researchers cannot directly use the raw data for location prediction.

The existing research methods usually divide location prediction into three steps: (1) preprocessing of the original trajectory data and screening out of meaningless and abnormal location points; (2) in the remaining location points, important locations with special significance to users are constructed according to point density, visit frequency and other information, such as the user’s home address, work unit and other areas inseparable from their daily life; (3) according to the training model of user’s historical access information to location points, the next location of users can be predicted given the current and historical location points of users. In terms of trajectory preprocessing and important locations construction, only articles using GPS data and check-in data as experimental data contains these two steps, articles using Wi-Fi location data directly take each access point as an important location. Similarly, articles using data recorded by base stations also take each base station as an important location, while articles using data recorded by traffic bayonets take each traffic bayonet as an important location.

Ashbrook et al. directly used a K-means algorithm to cluster location points to get important locations without utilizing any preprocessing method, and a Markov model was created for each important location, with transitions to every other location. Each node in the Markov model was an important location, and a transition between two nodes represented the probability of the user traveling between those two important locations [29]. Yang et al. preprocessed the trajectory data by setting the gradient threshold to filter noise points, then used the DBSCAN algorithm to cluster the remaining location points and output the clusters as important locations [30]. Montoliu et al. used a time-based clustering algorithm to preprocess the trajectory data, defined the processed location points as the stay points, and then used the grid-based clustering algorithm to extract important locations and defined them as the stay regions [31]. Killijian et al. directly used a density clustering algorithm called DJ-Cluster to extract important locations in the original trajectory data, in view of the shortcomings of the standard Markov model, they proposed an extended mobility Markov chain called *n*-MMC to improve the prediction accuracy [32]. Gidófalvi et al. used a grid-based approach to preprocess the original trajectory data, then periodically extracted and managed important locations that the user frequently visited, finally, they proposed an inhomogeneous continuous-time Markov model to predict when the user will leave his current region and where he will move next [33]. Yu et al. regarded the locations of each AP in the WLAN area where the user is located as important locations, and a step-2 Markov model was proposed to solve the state space expansion problem in high-order Markov predictor [34]. To solve the problem of the low prediction power of the standard Markov model and the problem of it being hard to determine the order of higher order Markov model in practice, Lv et al. presented a location prediction approach based on a novel adaptive multi-order Markov model, the approach first processed the raw location information of a user based on regular shape abstraction, and automatically determines the order of the model [35]. Chen et al. used the location points recorded by the traffic jam as experimental data set, they proposed the Global Markov Model and the Personal Markov Model to solve the sparse problem of personal trajectory data [21]. Rodriguez-Carrion et al. proposed a novel version of the LZ-based algorithm to perform location prediction on mobile devices [36]. Lu et al. established a Markov-based predictive model and used the trained model for location prediction of the model user [16]. Gunduz et al. first meshed the target user’s activity area and then uses artificial neural networks to predict the target user’s next location [4]. Reza et al. proposed a pipeline of three algorithms. First, they introduced a spatio-temporal event detection algorithm. Then, they introduced a clustering algorithm based on mobile contextual data. Their spatio-temporal clustering approach could be used as an annotation on raw sensor data [37].

Most researchers have used Markov-based models due to their good temporal trajectory data representation capability, while other researchers have attempted to deal with the location prediction problem using Hidden Markov models [38], Gaussian mixture models [39], Kalman filters [40] and so on. However, existing research methods still have some shortcomings in the three aspects of trajectory preprocessing, important locations extraction, and construction of prediction model. In particular, we have proposed an improved density clustering algorithm for extracting important locations [41]. Based on this, the paper will improve the trajectory preprocessing method and trajectory prediction model.

First, in the aspect of trajectory preprocessing, most researchers removed the trajectory outlier points by setting a time threshold or velocity threshold. Although this method is simple, it is difficult to set an appropriate threshold. Therefore, this paper first filters out the obvious noise points by setting a lower speed threshold, and then further extracts the feature points based on the structural changes of the trajectory.

Second, many researchers extracted important locations by using density clustering algorithms or grid clustering algorithms, however, the existing density clustering algorithms and grid clustering algorithms are too sensitive to the input parameters. Therefore, this paper an improved density peak clustering algorithm to extract important locations of users more accurately.

Third, existing trajectory prediction models often have the disadvantage of insufficient utilization of historical trajectory information, this paper uses a boosting algorithm to improve the predictive performance of the Markov model.

The remainder of this paper is organized as follows: the proposed approach and algorithm details are presented in the section “Proposed Framework”. The data set source and experimentation results are presented in the section “Experiment”. The conclusions of our work are presented in the “Conclusions” section.

## 2. Proposed Framework

**Figure 1:**
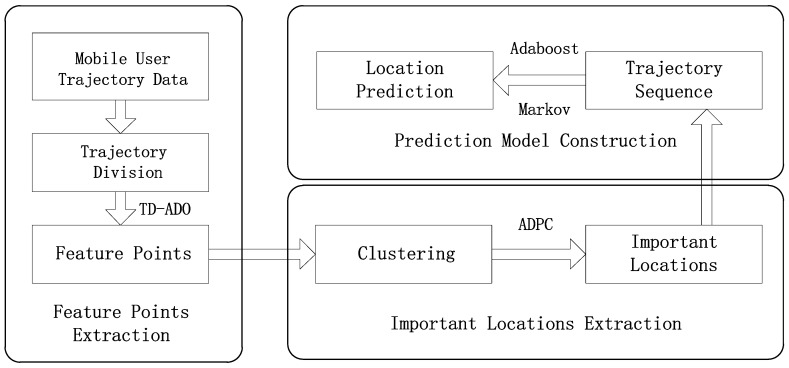
The framework of the proposed approach for the next location prediction of mobile user.

In this section, we describe in detail how the proposed framework extracts feature points and important locations and predict the next location. Figure 1 illustrates the overall training process of the proposed framework that consists of three steps to achieve the goal of predicting the next location of mobile user.

The training process proceeds as follows: First, a novel trajectory division method based on angle and distance offset for preprocessing trajectory data, named TD-ADO, is proposed to extract feature points and filter noise points.

Next, an improved density peak clustering algorithm named ADPC is proposed for extracting important locations from trajectory data sets filtered by TD-ADO. ADPC applies the theory of information entropy to the parameter decision of the density peak clustering algorithm, and help to determine the number of cluster centers according to the slope change trend.

Finally, the original trajectory data is converted into trajectory sequences consisting of important locations, which will be used for prediction model training. A fusion Markov with different order based on Adaboost algorithm named Adaboost-Markov model is proposed for location prediction, the weight coefficients of the training trajectory data and of each order Markov model are reasonably adjusted according to the prediction error rate, in this way, the advantages of each order model are fully leveraged and the information implicit in the prefix trajectory data is fully utilized.

### 2.1. Trajectory Preprocessing Algorithm

The purpose of trajectory division is to extract trajectory sampling points with large changes in user behavior, and attribute them to the data set of feature points. Each feature point serves as the end point of the previous sub-trajectory and the beginning of the next sub-trajectory, thus we can divide a complete trajectory into several continuous sub-trajectories by this method. 

In order to describe our algorithm better, this paper uses the definition of the trajectory in [42]. Let *TD* be a trajectory dataset, and the dataset consists of *n* trajectories, namely *TD* = {*TJ*_1_, *TJ*_2_, …, *TJ_n_*}. The trajectory (*TJ*) is a sequence composed of a number of trajectory sampling points in chronological order, namely *TJ_i_* = {*P*_1_, *P*_2_, …, *P_m_*}(1 ≤ *i* ≤ *n*). Among them, *P_j_*(1 ≤ *j* ≤ *m*) is a trajectory sampling point composed of latitude and longitude and sampling time, namely, <*Lat_j_*, *Lng_j_*, *T_j_*>.

**Definition** **1.** *(sub-trajectory). The sub-trajectory of the trajectory TJ_i_ is expressed as STJ_i_ = {P_s1_, P_s2_, …, P_sk_} (1 ≤ s1 < s2 < … < sk ≤ m).*

The sub-trajectories mentioned in this paper are all continuous, that is, from *P_s_*_1_ to *P_sk_* are continuous trajectory sampling points.

**Figure 2:**
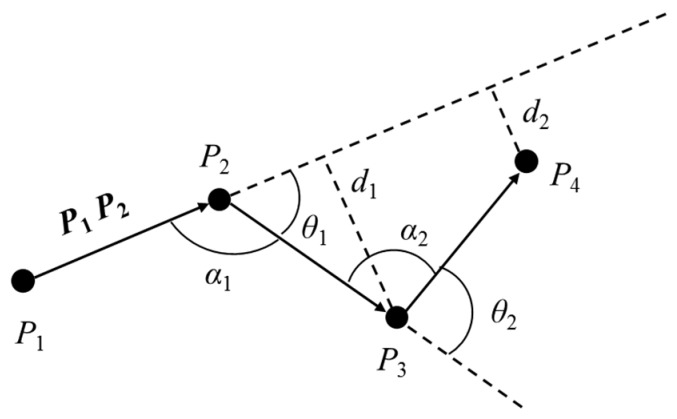
The angle and distance offset.

**Definition** **2.** *(angel offset). As shown in Figure 2, taking the trajectory TJ_i_ as an example, P_1_ is the initial trajectory point and **P_1_P_2_** is the initial movement behavior. The angle offset refers to the angle θ_i_ between the movement behavior **P_i_P_i+1_** and **P_i+1_P_i+2_** (1 ≤ i ≤ m−2) when they are connected at the end. For example, the angle θ_1_ between the movement behavior **P_1_P_2_** and **P_2_P_3_** when they are connected at the end and the angle θ_2_ between the movement behavior **P_2_P_3_** and **P_3_P_4_** when they are connected at the end.*

In this paper, the clockwise rotation angle is set as a positive value and the counterclockwise rotation angle is set as a negative value, i.e., *θ*_1_ is a positive value and *θ*_2_ is a negative value. In order to calculate the magnitude of the angle offset, the distance between two points must first be calculated from the latitude and longitude of the trajectory sampling point. The specific formula is defined in Equation (1):

(1) $$\begin{array}{c} A=\sin^{2}(\frac{Lat_{j}-Lat_{i}}{2})\\ B=\cos(Lng_{i})\cos(Lng_{j})\sin^{2}(\frac{Lng_{j}-Lng_{i}}{2})\\ d(P_{i},P_{j})=2R\arcsin\sqrt{A+B} \end{array}\}$$

*d*(*P_i_*, *P_j_*) is the distance between the trajectory sampling points *P_i_* and *P_j_*, and *R* is the radius of the earth. The trajectory angle offset function is defined in Equations (2) and (3):

(2) $$\alpha_{i}=\arccos\frac{\left({\left|P_{i}P_{i+1}\right|}^{2}+{\left|P_{i+1}P_{i+2}\right|}^{2}-{\left|P_{i}P_{i+2}\right|}^{2}\right)}{2{\left|P_{i}P_{i+1}\right|}^{2}{\left|P_{i+1}P_{i+2}\right|}^{2}}$$

(3) $$\theta_{i}=\left\{ \begin{array}{l} \alpha -\pi ,P_{i}P_{i+1}\times P_{i+1}P_{i+2}>0\\ \pi -\alpha ,P_{i}P_{i+1}\times P_{i+1}P_{i+2}\geq 0 \end{array}\right.$$

**Definition** **3.** *(distance offset). The distance offset is the vertical distance from the trajectory sampling points to the line where the initial movement is located.*

As shown in Figure 2, the vertical distances *d*_1_ and *d*_2_ from the sampling points *P*_3_ and *P*_4_ to the extension line of initial movement are the distance offsets. The trajectory distance offset function is defined in Equation (4):

(4) $$d_{i}=\left|P_{2}P_{i+2}\right|\sin∠P_{1}P_{2}P_{i+2}$$

For the trajectory *TJ_i_*, firstly, from the sampling point *P*_3_, calculate the angle offset *θ*_1_ and the distance offset *d*_1_, if the absolute value of *θ*_1_ does not exceed the angle offset threshold *θ_th_*, and *d*_1_ does not exceed the distance offset threshold *d_th_*, then continue to calculate the angle and distance offset of the sampling point *P*_4_, and so on.

If the angle offset or distance offset of the sampling point *P_i_* exceeds the corresponding threshold, *P_i_* will be assigned to the data set of feature points, and define ***P_i_P_i_*_+1_** as the new initial movement behavior, continue to perform the above steps from the sampling point *P_i_*_+2_. Finally, the trajectory *TJ_i_* will be divided into several continuous sub-trajectories. The time complexity of the trajectory dividing algorithm in this paper is *O*(*n*), where *n* is the number of trajectory sampling points.

**Figure 3:**
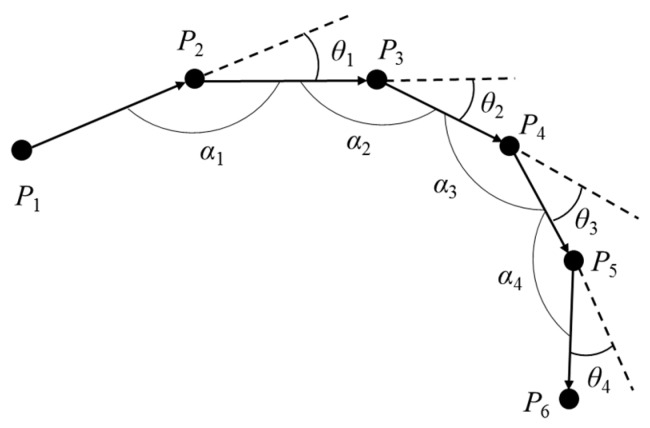
The continuous clockwise rotation trajectory.

**Figure 4:**
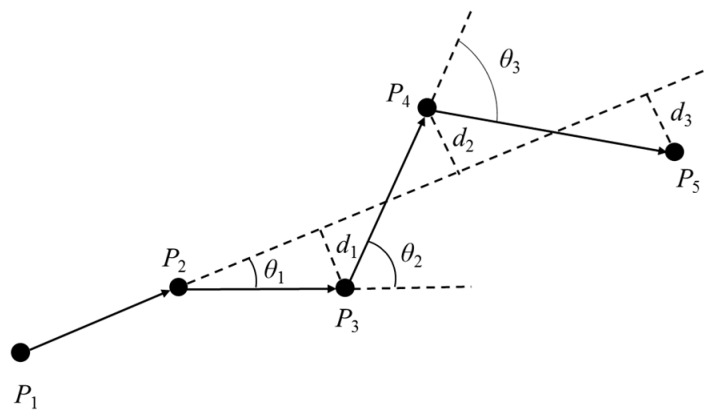
The regular clockwise-anticlockwise rotation trajectory.

The trajectory division algorithm using the angle and distance offset can effectively avoid the following two conditions: (1) If the trajectory is divided only by setting the angle offset threshold value, the feature points in Figure 3 cannot be extracted. Each angle offset in Figure 3 is small, but the shape of the *P*_1_~*P*_6_ trajectory segment is close to a semicircle due to the stacking effect of multiple clockwise rotations. Obviously, it is obviously unreasonable not to classify the six sampling points *P*_1_~*P*_6_ as feature points, which will lead to the omission of important locations. (2) If the trajectory is divided only by setting the distance offset threshold, missed selection of important locations may also occur. In Figure 4, since the trajectory has a regular clockwise and anti-clockwise rotation, the sampling points *P*_3_~*P*_5_ are all near the straight line where the initial movement behavior is located. However, the angle offsets between connected movements are already close to 90°, and it is obviously not appropriate to classify all sampling points of this trajectory segment as feature points.

The complete algorithm pseudo code is given below:

**Algorithm**: TD-ADO1: **Input**: *TJ_i_* = {*P*_1_, *P*_2_, …, *P_m_*}, angle offset threshold *θ_th,_* distance offset threshold *d_th_*; 2: **Output**: Feature Points FP; 3: *P*_1_→FP; 4: *i* = 1; 5: **repeat** 6: initial movement behavior = ***P_i_P_i_*_+1_**; 7: **for**
*j* = 1 to *m*−2 **do** 8:  **if**
*θ_j_* > *θ_th_* || *d_j_* > *d_th_*
**then** 9:   *P_j_*_+2_→FP; 10:   *i* = *j*+2; 11:   break; 12:  **else** 13:   **if**
*j* = *m*−2 **then** 14:    *P_j_*_+2_→FP; 15:   **end if** 16:  **end if** 17: **end for** 18: **until**
*P_m_*→FP.19: **return** FP.

The algorithm adds the initial sampling point and the final sampling point of each trajectory to the feature points, because the starting point and ending point of the trajectory usually have special meanings. For example, when a user turns on the mobile phone or turns off the airplane mode every day when he goes out, the starting point of the trajectory is often the location of the user’s residence. Similarly, when the user turns off the mobile phone or turns on the flight mode before going to bed at night, the trajectory record of the day is terminated. In this way, the trajectories in the trajectory set are divided according to the above algorithm, and each trajectory is divided into several sub-trajectories, and the extracted feature points form a data set.

### 2.2. The Important Locations Extraction Algorithm

Researchers often cluster trajectory sampling points based on their density to extract important locations. However, commonly used density clustering algorithms usually have the shortcomings that the clustering result is too sensitive to the input parameters. How to determine the appropriate input parameters has become an urgent problem to be solved. In this paper, we propose an improved density peak clustering algorithm called ADPC to cluster the feature points and extract the important locations. The density peak clustering algorithm was a new density clustering algorithm proposed by Rodriguez et al. [43]. Compared with the classical density clustering algorithms, the algorithm is simple in principle, requires only one input parameter, and does not require iteration, and it can cluster data of various shapes. However, it is necessary to select the clustering center manually through the decision graph, which not only increases the redundancy of the algorithm, but also causes subjective troubles. The improved density peak clustering algorithm ADPC incorporates the self-adaptive improvement of the cut-off distance and the clustering center, so that it can automatically determine the best input parameters and clustering center according to the distribution of sampling points. 

The only input parameter of the density peak clustering algorithm is the cut-off distance *d_c_*, this paper adopts the percentage to represent the cut-off distance *d_c_*, arrange the distances between all two data points in ascending order, and take the distance value corresponding to a certain percentage in the distance set.

In this model, for a data set *S* = ${\left\{x_{i}\right\}}_{=1}^{NI}$ to be clustered, there are two quantities that need to be calculated. One is the local density of each data point *ρ_i_*, and the other is the minimum distance between each data point and the higher density point *δ_i_*.

The formula for the local density is given by Equation (5):

(5) $$\rho_{i}=\sum_{j}e^{-{\left(\frac{d_{ij}}{d_{c}}\right)}^{2}}$$

*δ_i_* is the minimum distance from data point *i* to data point *j* with a higher local density, defined in Equation (6):

(6) $$\delta_{i}=\underset{\rho_{i}<\rho_{j}}{\min}(d_{ij})$$

For the data point *k* with a global maximum density, *δ_i_* is defined as the maximum distance between the data point and the remaining data points.

**Figure 5:**
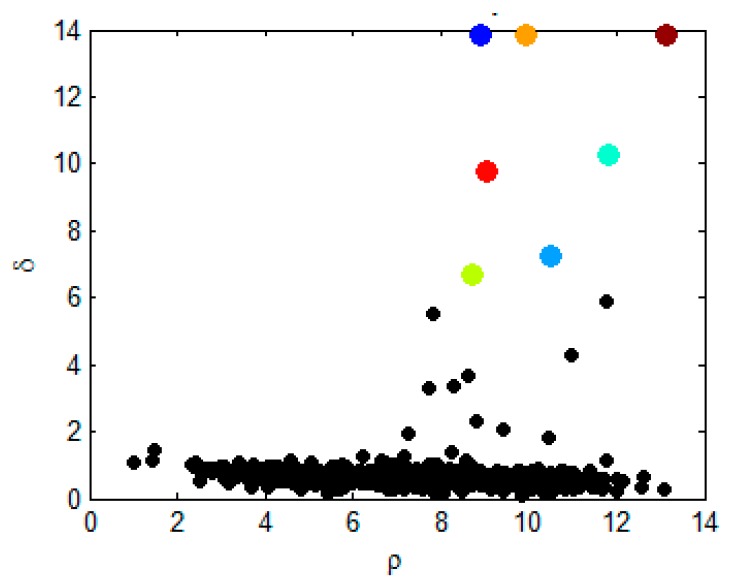
Decision graph.

At this point, for each data point in the data set *S*, its local density *ρ_i_* and the minimum distance *δ_i_* can be calculated. The density peak clustering algorithm regards the data points that have large *ρ_i_* and *δ_i_* at the same time as the cluster centers, and the remaining data points are allocated to clusters that are closest to the nearest cluster centers. As shown in Figure 5, the horizontal axis represents the local density of each point, the vertical axis represents the minimum distance of each point, and the color points are the points selected as the cluster centers.

The density peak clustering algorithm mainly has two shortcomings. On the one hand, there is no reliable reference for the selection of its input parameter *d_c_*. The author of this algorithm, Rodriguez, gave the recommendation to select the top 1% to 2% of the cut-off distances after the distance between all data points was sorted in ascending order. However, for a data set with a disproportionately different size and distribution, this general method of parameter selection will often lead to poor results. On the other hand, the density peak clustering algorithm relies on the distribution of points on the decision graph to select the cluster center, which is too subjective.

The ADPC algorithm is an improved algorithm in this paper. Its essence is to achieve the data set clustering through the automatic selection of the cut-off distance and the clustering centers.

Rodriguez gave an idea for reference, that is, consider the product of *ρ_i_* and *δ_i_*:

(7) $$\gamma_{i}=\rho_{i}\delta_{i}$$

First, *ρ_i_* and *δ_i_* are normalized, then *γ_i_* is obtained for each data point, and arrange them in descending order. The larger the *γ_i_*, the more likely it is to be a cluster center.

To visually show the relationship between *d_c_* and *γ_i_*, Equation (7) can also be expressed as:

(8) $$\gamma_{i}=\delta_{i}\sum_{j}e^{-{\left(\frac{d_{ij}}{d_{c}}\right)}^{2}}$$

Finding the minimum value of the information entropy based on *γ_i_* to achieve the adaptive cut-off distance and obtaining the maximum value of the slope change trend to achieve automatic selection of cluster centers is the focus of this paper.

This paper proposes a cut-off distance adaptive method based on the minimization of information entropy. In information theory, entropy is used as a measure of system uncertainty. The greater the entropy, the stronger the uncertainty of the system. The formula for calculating information entropy is as follows:

(9) $$H=-\sum_{i=1}^{n}p_{i}\log p_{i}$$

Similarly, apply the definition of entropy to this algorithm, replace the probability value in the information entropy formula with *γ*, that is, the larger the *γ*, the more likely it is that the corresponding data point is the cluster center. If the points in the dataset have the same *γ*, the uncertainty of the system is the greatest, and the difficulty of determining the cluster center is also highest at this time. On the other hand, if the entropy value is the minimum, the distribution difference of *γ* is the most obvious, and it is the easiest to determine the cluster center.

Combining Equations (8) and (9), this paper gives the relationship between cut-off distance *d_c_* and entropy *H* with Equation (10):

(10) $$H=-\sum_{i=1}^{n}\left(\delta_{i}\sum_{j}e^{-{\left(\frac{d_{ij}}{d_{c}}\right)}^{2}}\right)\log(\delta_{i}\sum_{j}e^{-{\left(\frac{d_{ij}}{d_{c}}\right)}^{2}})$$

Let the cut-off distance *d_c_* gradually increase from zero, find *d_c_* that makes the entropy value *H* the minimum, and use it as the optimal cut-off distance for the next clustering, so that the cut-off distance is self-adaptive.

**Figure 6:**
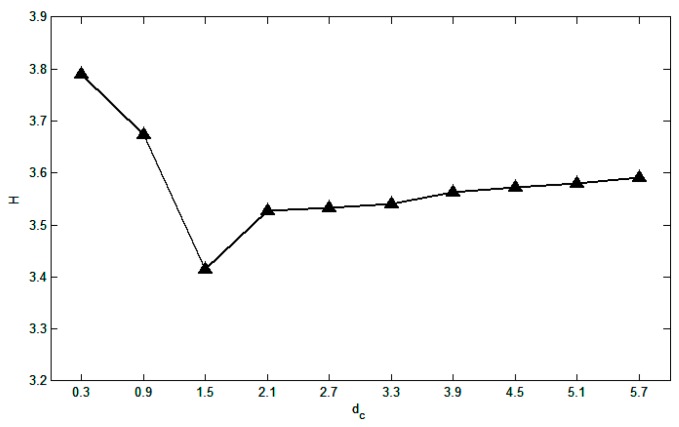
The trend of information entropy under different cut-off distances.

Take the data set of Ref. [43] as an example and get the result shown in Figure 6. The horizontal axis indicates the cut-off distance *d_c_*, and the vertical axis indicates the entropy value *H*. It can be seen from the figure that when the entropy value *H* reaches the minimum, the cut-off distance *d_c_* is 1.5%.

**Figure 7:**
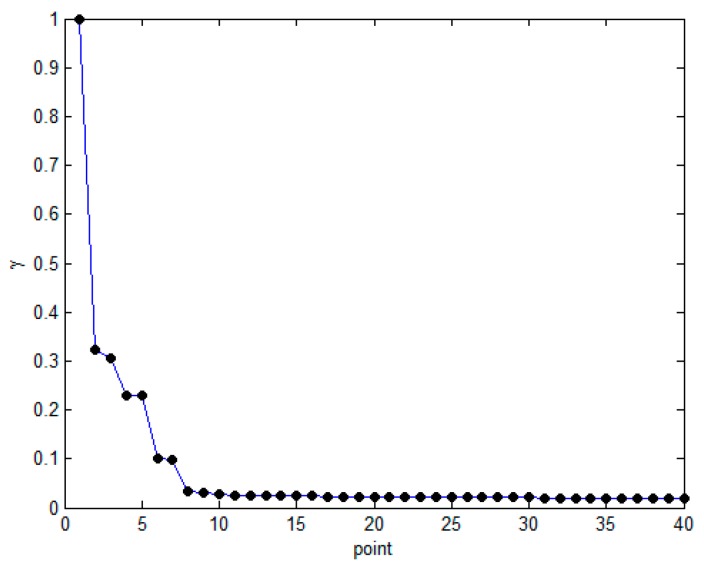
*γ_i_* sort graph.

Taking the 2000 random sample points given in Ref. [43] as an example, we select the first 1.5% *d_c_*, calculate the first 40 *γ_i_*, and then use MATLAB to generate a decision map based on the results. The results are shown in Figure 7. The first five points are the actual cluster centers, and there is a steep downward trend from the cluster center to the first non-cluster center. Therefore, this paper uses the trend of slope change to automatically select the cluster center and define this trend as *tend_i_*:

(11) $$tend_{i}=(i-1)\frac{\gamma_{i-1}-\gamma_{i}}{\gamma_{i}-\gamma_{i+1}}$$

Considering that there may be a tendency similar to the slope change from point 1 to point 2 in Figure 7, the weight *i*−1 is introduced for *tend_i_*. However, as the number of data points increases, the weight *i*−1 may cause *tend_i_* to increase rapidly, resulting in erroneous judgments.

**Figure 8:**
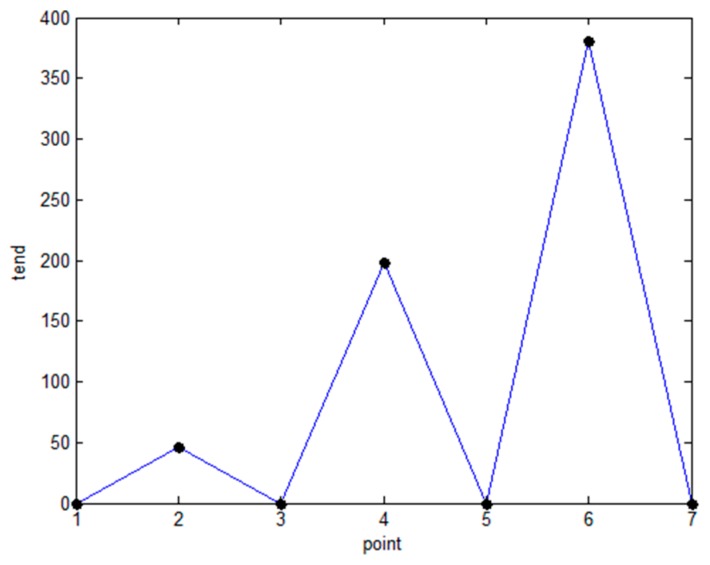
Slope change trend graph.

In this regard, the paper uses the average value of all *γ_i_* in the ranking graph as the threshold *θ* to solve the problem that the weight will cause the rapid increase of *tend_i_*. First, the data points whose *γ_i_* is larger than the threshold *θ* are selected from 40 data points, and *tend* of the selected data point is calculated. As shown in Figure 8, after the screening, there are still seven data points remaining. It can be seen that the *tend* at the sixth point is the largest, so the first five points are selected as potential cluster centers. The density peak clustering algorithm can well classify the data points with very small *ρ_i_* and large *δ_i_* into outliers. However, relying solely on the product of *ρ_i_* and *δ_i_* to select cluster centers may result in false selection of cluster centers: For the point with large *ρ_i_* and small *δ_i_*, that is, two high density points within the same cluster are likely to be mistakenly selected as clustering centers and the cluster is divided. Therefore, the optimal *d_c_* obtained by Equation (10) is set as the distance threshold of the potential cluster center, and the algorithm is prevented from erroneously judging the point with large *ρ_i_* and small *δ_i_* as the cluster center.

The concrete steps of ADPC are as follows:

Step 1. Calculate the product *γ_i_* of normalized *ρ_i_* and *δ_i_* for each data point.
Step 2. Find *d_c_* that minimizes the entropy.
Step 3. Sort *γ_i_* of each data point in descending order, and select the appropriate number of points according to the total number of points in the data set and generate sorting charts in descending order.
Step 4. Calculate the average value *θ* of *γ* in the *γ_i_* sort diagram and use *θ* as the threshold to filter out data points with *γ_i_* greater than *θ*.
Step 5. According to the slope change trend formula, the *tend* of the filtered data points is obtained.
Step 6. The *i*−1 data points before the data point *i* with the largest tend are regarded as potential cluster centers.
Step 7. First, use the first data point as the actual cluster center to determine whether the distance between the second data point and its distance is less than the distance threshold *d_c_*. If it is less than, then the second data point is treated as a non-clustering center; otherwise, use the second data point as the actual clustering center.
Step 8. By analogy, determine whether the distance between the *k*th potential cluster center and all actual cluster centers are greater than the distance threshold, and then treat it as the actual cluster center or non-cluster center according to the judgment result.
Step 9. Finally, according to all the selected actual cluster centers, cluster the remaining data points.

**Figure 9:**
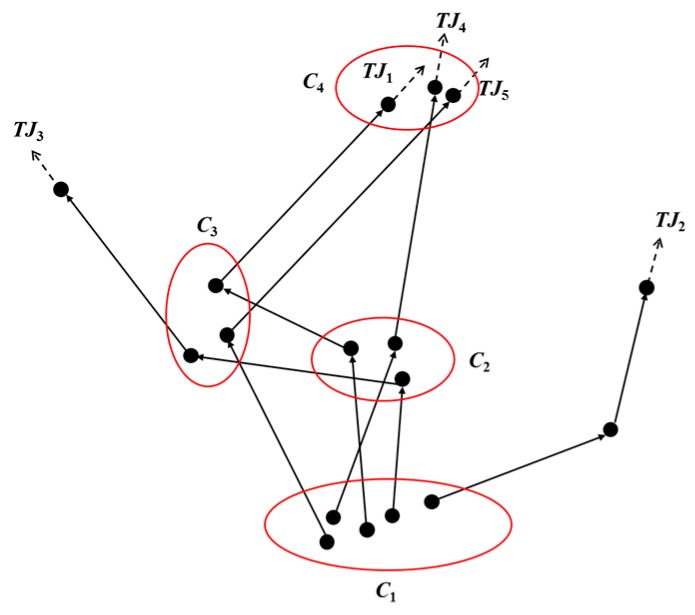
The diagram of important location clusters.

Figure 9 illustrates the process of clustering important points from points of feature, there are five simplified trajectories consists of feature points, after clustering the points of feature extracted by the TD-ADO algorithm, four clusters *C*_1_ to *C*_4_ are formed, and the feature points that have not been red circled are judged as noise points. In this way, the important locations consist of all sampling points in the red circle.

### 2.3. Locations Prediction Model

In order to facilitate the understanding of the following, this paper gives the following definitions.

**Definition** **4.** *(Markov chain). Suppose there is a random process {X_n_,n ∈ T}, and there are finite states i_0_,i_1_, …, i_n_ ∈ I, if P{X_n+1_ = i_n+1_|X_0_ = i_0_, X_1_ = i_1_, …, X_n_ = i_n_} = P{X_n+1_ = i_n+1_|X_n_ = i_n_}, then {X_n_, n ∈ T} is a Markov chain, that is, the next state of the object is only related to the state at this time, also called the first-order Markov chain. If the next state of the object is not only related to the state at this time, but also related to the first k-1 states, it is called a k-order Markov chain.*

**Definition** **5.** *(Trajectory sequence): The user’s trajectory sequence is a simplified representation of its original trajectory after trajectory division and density clustering, and is composed of extracted important locations. For example, the original trajectory of the user is expressed as TJ_i_ = {P_1_, P_2_, …, P_m_}(1 ≤ i ≤ n), and the processed simplified trajectory is expressed as TS_i_ = {C_k1_, C_k2_, …, C_kj_}(1 ≤ i ≤ n).*

**Definition** **6.** *(Prefix trajectory sequence): Suppose that given a trajectory sequence TS_i_ = {C_k1_, C_k2_, …, C_kj_}(1 ≤ i ≤ n), and there is 1 < l < j, to predict which important location C_kl_ is, then {C_k1_, C_k2_, …, C_k(l−1)_} is its corresponding prefix trajectory sequence, whose elements are called prefix trajectory elements.*

**Definition** **7.** *(Trajectory Markov chain): The state space of the trajectory Markov chain is composed of the user’s important locations C_1_, C_2_, …, C_n_.*

If the user’s next important location depends on the current location and the past *k*-1 locations, the Markov chain is called a *k*-order trajectory Markov chain, namely:

(12) $$C_{p}=\underset{C_{n+1}}{\arg\max}\{p(C_{n+1}|C_{n-k+1},C_{n-k+2},\ldots ,C_{n})\}$$

where *C_p_* represents the prediction result of the user’s next important location, and *C_n_* represents the current important location of the user.

**Figure 10:**
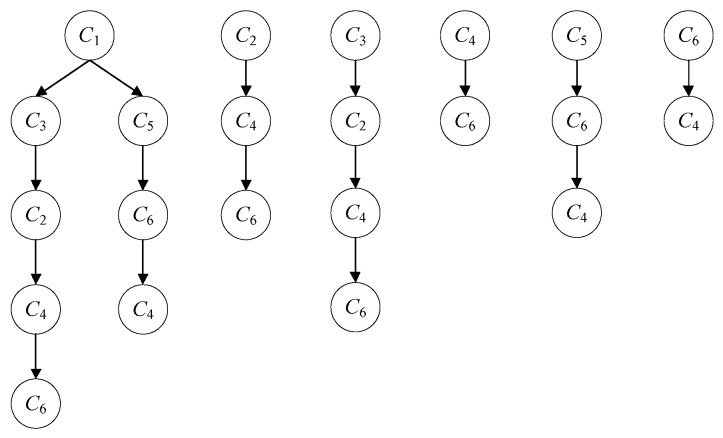
User historical trajectory sequence pattern tree.

The low-order Markov model has the advantages of small time cost and fast calculation speed, but its prediction accuracy is not high enough. Although the higher-order Markov model has higher prediction accuracy, the spatial state expansion problem makes it difficult to apply to the large-scale trajectory data set, and some researchers [35] have pointed out that there is a limit to the prediction performance growth caused by the increase of the order. If the user’s historical trajectory data is difficult to match the Markov model with high-order, the prediction performance of the model will be reduced due to the sparse rate. As shown in Figure 10, taking a historical trajectory pattern tree of a user as an example, it is assumed that a third-order Markov model is adopted, and the prefix trajectory sequence is *C*_2_→*C*_4_→*C*_5_, and the next important location of the user is predicted based on the mobile pattern tree. However, there is no historical trajectory with *C*_2_→*C*_4_→*C*_5_ as the prefix sequence in this pattern tree, and the third-order Markov model cannot make prediction. Moreover, when the order of the Markov model is high, even if a prefix trajectory sequence as *C*_2_→*C*_4_→*C*_5_ is found in the user historical trajectory sequence pattern tree, there is a high possibility that the number of matches is too small and the sparse rate is still high, which may lead to poor prediction result.

In view of the above problems, the first-order Markov model only utilizes the current interest region of the user, and the utilization rate of the historical trajectory data is not high; while the high-order Markov model has problems of difficulty in matching and high sparse rate, the historical trajectory data cannot be fully utilized as the same. Therefore, combining low-order models with high-order models is a feasible method. Then, reasonably determining the influence coefficient of each model with different order is an important prerequisite for ensuring good prediction performance. From a macro perspective, high-order Markov model usually has higher prediction accuracy, and should have greater “discourse power” in the fusion model. In order to determine the specific influence coefficient of each model with different order, a mobile user location prediction method based on Adaboost-Markov model is proposed in this paper. As a representative lifting method, Adaboost algorithm can combine a plurality of weak classifiers by changing the probability distribution of training data and the weight coefficient of weak classifier to generate a strong classifier [44]. In this paper, the fusion model order *k* is adaptively determined, and the first-order to *k*th-order Markov model are used as *k* weak predictors. The probability distribution of user trajectory data and the weight coefficients of each Markov model are changed by Adaboost algorithm, and finally a multi-order fusion Markov is generated, which is used to predict user important location.

**Figure 11:**
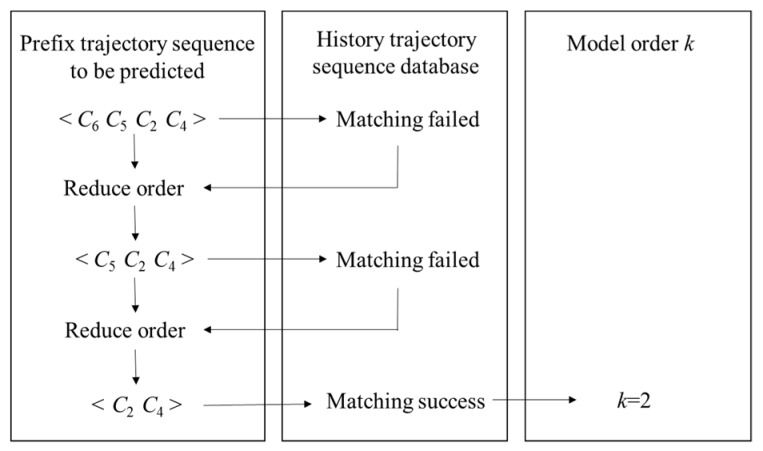
Example of adaptive determination of order *k*.

The order *k* is determined by the maximum matching step length of the user prefix trajectory sequence and the historical trajectory sequence. Taking Figure 11 as an example, the user’s original trajectory data is preprocessed to establish a historical trajectory sequence database. The user determines the number of elements of the prefix trajectory sequence, and inputs the prefix trajectory sequence to be predicted. If the same sequence as the prefix trajectory sequence can be found in the historical trajectory sequence, the matching is successful, and the number of prefix trajectory elements is set to the model order *k*; otherwise, if the matching fails, the order is automatically reduced, remove the element from the prefix trajectory sequence that is furthest from the current state, and continue to seek a match in the historical trajectory sequence database, repeat the above steps until the match is successful.

After the model order *k* is determined, the first-order to *k*th-order Markov model is merged, that is, the Adaboost-Markov model is composed of *k m*-order (*m* = 1, 2, …, *k*) Markov models, The weight coefficient *α_m_* of each model is negatively correlated with the prediction error of itself, the larger the prediction error, the smaller the weight coefficient of the corresponding model, and vice versa. For training trajectory sequences with *N* elements, the prediction error *e_m_* of the *m*-order (*m* = 1, 2, …, *k*) Markov model is calculated as follows:

(13) $$e_{m}=\sum_{i=1}^{N}w_{mi}I_{m}^{i}$$

where *w_mi_* is the weight of the training samples corresponding to the *m*-order (*m* = 1, 2,…, *k*) Markov model, which is initially set to 1/*N*, and $\sum_{i=1}^{N}w_{mi}=1$. $I_{m}^{i}$ is the predictor of the *m*-order Markov model for the training sample *i*, and its formula is as shown in Equation (14):

(14) $$I_{m}^{i}=\left\{ \begin{array}{l} 1,error\;prediction\\ 0,correct\;prediction \end{array}\right.$$

After calculating the prediction error *e_m_*, the model weight coefficient αm can be calculated as follows:

(15) $$\alpha_{m}=\frac{1}{2}\log\frac{1-e_{m}}{e_{m}}$$

Then, the weight coefficient of the *m*-th order model obtained by the above method is used to update the weight of the training sample, and the weight *w_m_*_+1,*i*_ of the training sample corresponding to the *m*+1 order (*m* = 1, 2, …, *k*−1) Markov model is calculated, improve the sample weight of mispredicted in the *m*-order Markov model and reduce the weight of sample with the correct prediction result, so as to make the mispredicted samples get more attention in the next round of learning. The calculation method is as shown in Equations (16) and (17):

(16) $$w_{m+1,i}=\frac{w_{mi}}{Z_{m}}\exp(\alpha_{m}I_{m}^{i})$$

(17) $$Z_{m}=\sum_{i=1}^{N}w_{mi}\exp(\alpha_{m}I_{m}^{i})$$

where *Z_m_* is the normalization factor, which makes *w_m_*_+1,*i*_ a probability distribution. Then, the prediction error *e_m_*_+1_ and the model weight coefficient *α_m_*_+1_ are calculated according to the new training sample weight and the *m*+1 order Markov model, and so on, until the prediction error *e_k_* of the *k*-order Markov model and the model weight coefficient *α_k_* are calculated. Finally, normalize the weight coefficients of each order model, its formula is as follows:

(18) $$\beta_{m}=\frac{\alpha_{m}}{\sum_{m=1}^{k}\alpha_{m}}$$

The Adaboost-Markov model can be expressed as follows:

(19) $$G(x)=\sum_{m=1}^{k}\beta_{m}G_{m}(x)$$

Among them, *G_m_*(*x*) represents the *m*-order Markov model.

## 3. Experiments

In this section, in order to verify the clustering performance of the ADPC algorithm, we first compare the accuracy, normalized mutual information (NMI) and F-measure of DBSCAN algorithm, density peak clustering algorithm and ADPC algorithm on the UCI dataset [45]. The data set information is shown in Table 1. Then the real mobile user trajectory dataset Geolife [9] is used to detect the prediction performance of our method, this data set information is shown in Table 2. The experimental environment is the Windows 10 64-bit operating system, Intel Core i7-6700HQ @ 2.60 GHz CPU, 8 G memory, using MATLAB2014a for simulation experiments. On the parameter selection of the trajectory preprocessing algorithm, *θ_th_* = 15°, *d_th_* = 3 m.

### 3.1. Clustering Performance

**Figure 12:**
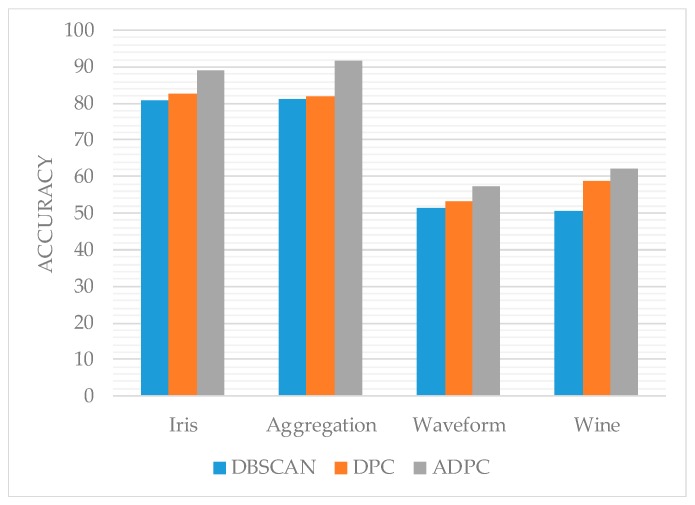
The accuracy of each algorithm.

**Figure 13:**
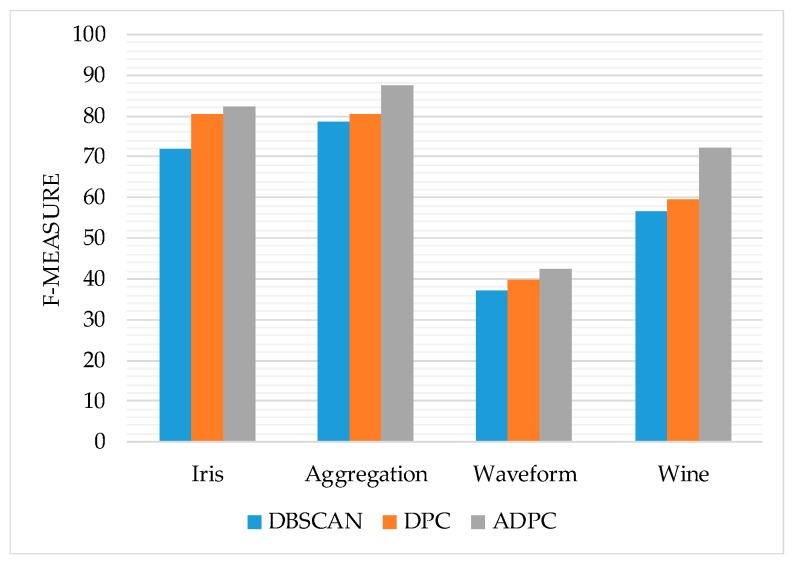
The F-measure of each algorithm.

**Figure 14:**
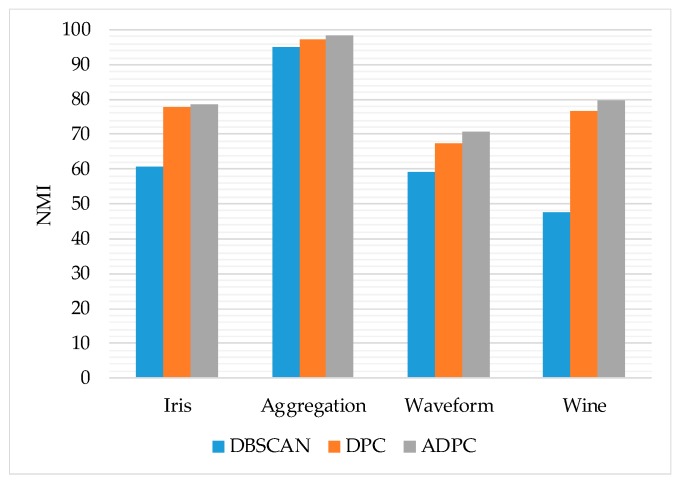
The normalized mutual information of each algorithm.

From Figure 12, Figure 13 and Figure 14, it can be seen that the trend of ADPC>DPC>>DBSCAN on the accuracy rate, F-measure, and normalized mutual information of the algorithm as a whole indicates to some extent the ADPC algorithm’s superior performance on clustering. 

However, the performance of the ADPC algorithm on the high-dimensional datasets such as Waveform and Wine is still not ideal. For the two-dimensional Aggregation dataset, the accuracy, F-measure value, and normalized mutual information of the ADPC algorithm have reached more than 90%, and the performance is good. Therefore, the ADPC algorithm can be used for two-dimensional location points.

### 3.2. Prediction Performance Analysis of Various Model

In this section, 90% of the Geolife trajectory data set are randomly selected for training models, and the remaining 10% of the trajectory data is used to detect prediction performance. Firstly, in order to verify the rationality of weight coefficient allocation using Adaboost algorithm, the prediction accuracy of the Adaboost-Markov model, the multi-order fusion Markov model with equal weight coefficients and the common Markov model under different prefix elements are compared.

**Figure 15:**
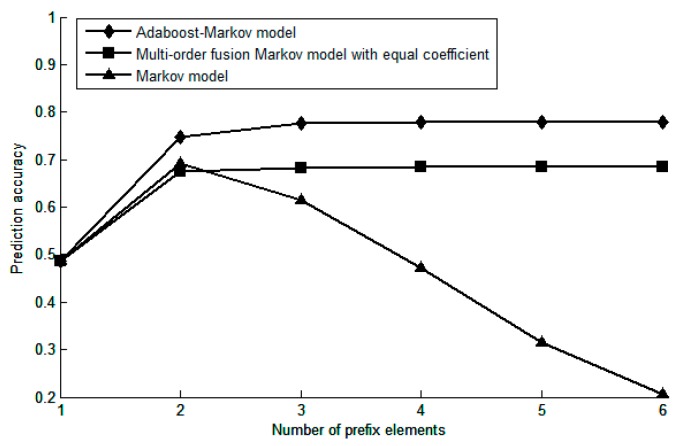
The relationship between the number of prefix elements and the prediction accuracy.

As shown in Figure 15, the horizontal axis represents the number of prefix elements, and the vertical axis represents the prediction accuracy. For the common Markov model, the number of prefix elements is equivalent to the model order, the Adaboost-Markov model and the multi-order fusion Markov model with equal weight coefficients adopt the adaptive method to determine the model order *k*. Therefore, with the increase of the number of prefix elements, the prediction accuracy of the common Markov model firstly shows an increasing trend, reaching a maximum at the second-order, and then gradually decreasing due to the increase of the matching sparse rate. The Adaboost-Markov model effectively overcomes this problem: he prediction accuracy first increases with the number of prefix elements, when the number of prefix elements is 3, the maximum is basically reached, then the prediction accuracy is stable near the maximum. 

Moreover, it can be seen from the figure that the weight coefficient of the model determined by the Adaboost algorithm is reasonable and effective, the prediction accuracy of the Adaboost-Markov model is higher than the multi-order fusion Markov model with equal weight coefficients regardless of the number of prefix elements in the given sequence to be predicted.

**Figure 16:**
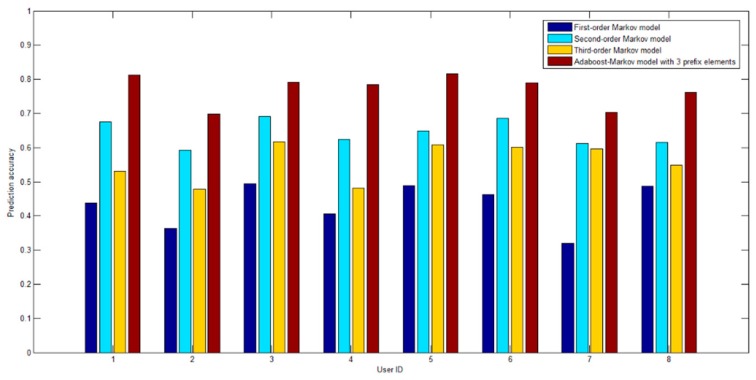
Prediction accuracy comparison under different trajectory data set.

In order to verify the universality of the model, this paper randomly selects the trajectory data of eight users from the Geolife data set, and compares the first to third-order Markov model and the Adaboost-Markov model under three prefix elements on different user trajectory data sets. As shown in Figure 16, the prediction accuracy of the Adaboost-Markov model is better than that of the first to third-order Markov model on different trajectory data sets. It can be seen that the proposed model does not depend on a specific data set and has good universality.

**Figure 17:**
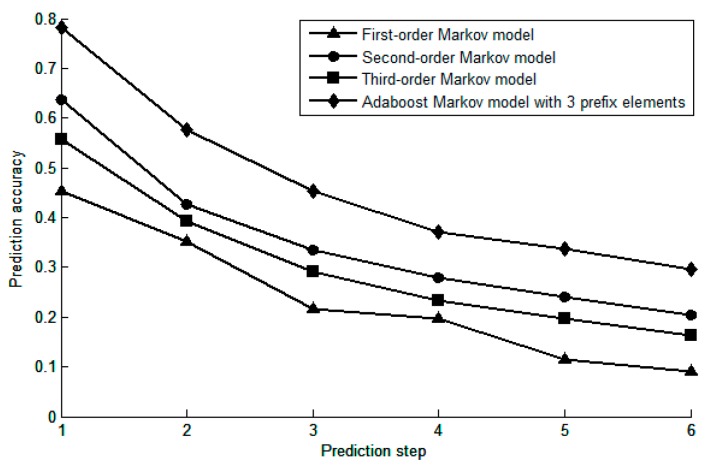
Relationship between prediction steps and prediction accuracy.

In the location prediction of mobile users, multi-step prediction ability is also one of the keys to reflect the prediction performance of the model, that is, predicting the region of interest where the user is after *n* steps. In order to verify the multi-step prediction ability of the model, the prediction accuracy of the Adaboost-Markov model with 3 prefix elements and the first to third -order Markov model are compared under different prediction steps, the experimental results are shown in Figure 17.

It can be seen from the figure that with the increase of the prediction step size, the prediction accuracy of each model decreases to some extent, but the prediction accuracy of the Adaboost-Markov model is better than the other three models. This is because the common Markov model directly uses a fixed-length step transition matrix when performing multi-step prediction, and has poor adaptability to the trajectory data. In this experiment, the Adaboost-Markov model selects three prefix elements, and its order *k* is flexibly changed in the first to third-order according to the specific trajectory data, which not only ensures that the time cost is not too large, but also makes the prediction accuracy is close to the maximum, so the prediction accuracy of the Adaboost-Markov model is relatively high.

## 4. Conclusions

This paper studies a location prediction method based on the Markov model for mobile user trajectory data. The method of combining trajectory division and density clustering is used to process trajectory data, the original trajectory data is transformed into a trajectory sequence composed of important locations, and the weight coefficient is determined for the multi-order fusion Markov model using the Adaboost algorithm. Experiments on the real user trajectory data set Geolife prove that the proposed method overcomes the low prediction accuracy of low-order Markov model and the high sparse rate of high-order Markov model to some extent, and makes full use of the user’s prefix trajectory information. The next step will take into account factors such as weather, time (working days and rest days) and user-related social data, in order to further improve the prediction accuracy of the model.

## Figures and Tables

**Table 1:** UCI dataset used in the experiment.

Data Set	Category	Sample	Dimension
Iris	3	150	4
Aggregation	7	788	2
Waveform	3	500	21
Wine	3	178	13

**Table 2:** Geolife dataset used in the experiment.

Data Collection Time	Number of Users	Number of Trajectories
2007.4~2012.8	182	17624
